# Access Pain During Transforaminal Endoscopic Lumbar Discectomy for Foraminal or Extraforaminal Disc Herniation

**DOI:** 10.3390/diagnostics14202337

**Published:** 2024-10-21

**Authors:** Yong Ahn, Ji-Eun Choi, Sol Lee

**Affiliations:** 1 Department of Neurosurgery, Kyung Hee University Hospital at Gangdong, Kyung Hee University College of Medicine, Seoul 05278, Republic of Korea; 2 Department of Neurosurgery, Gil Medical Center, Gachon University College of Medicine, Incheon 21565, Republic of Korea; ch6821@naver.com; 3 Basgenbio Research Institute, Seoul 04167, Republic of Korea; dlthf1028@naver.com

**Keywords:** diskectomy, endoscopy, hospitalization, lumbosacral region, operative time, pain, percutaneous procedure, spinal nerve roots

## Abstract

Background/Objectives: Transforaminal endoscopic lumbar discectomy (TELD) under local anesthesia is a promising minimally invasive surgical option for intractable lumbar disc herniation (LDH). However, our understanding of access pain prediction during foraminal pathological procedures is limited. To our knowledge, no predictive rules for access pain have been established during TELD for foraminal or extraforaminal LDH. This study, with its potential for predicting access pain during TELD and discussing strategies for pain prevention and management, could significantly benefit the field of endoscopic spine surgery. Methods: This observational study included 73 consecutive patients who underwent TELD for foraminal or extraforaminal LDH between January 2017 and December 2022. Preoperative clinical and radiographic factors affecting significant access pain and the impact of access pain on clinical outcomes were evaluated. Results: The rate of significant access pain was 13.70% (10 of 73 patients). Extraforaminal LDH tended to cause more severe pain than did foraminal LDH during TELD under local anesthesia (*p* < 0.05). Although the degree of access pain was not related to global clinical outcomes, increased pain was strongly associated with prolonged operative time and length of hospital stay (*p* < 0.05). Conclusions: TELD could be an effective surgical option for foraminal or extraforaminal LDH under local anesthesia. More access pain might develop during TELD for extraforaminal LDH. The extraforaminal component of LDH could narrow the safe working zone. Significant access pain might prolong the duration of surgery and hospitalization. Thus, a specialized technique is required for the clinical success of TELD.

## 1. Introduction

Lumbar disc herniation (LDH) is a common condition that affects millions worldwide, leading to substantial pain and disability. The development of minimally invasive surgical techniques, such as transforaminal endoscopic lumbar discectomy (TELD), has revolutionized LDH treatment [1,2,3,4,5,6]. The effectiveness of TELD has been validated in several randomized controlled trials [7,8,9,10] and meta-analyses [11,12,13,14,15,16,17,18,19]. TELD can be performed using a percutaneous posterolateral endoscopic approach under local anesthesia. However, access pain during the transforaminal approach poses a significant technical challenge during this minimally invasive procedure [20,21,22], primarily attributed to the exiting nerve root (ENR) and other neural irritations. Various studies have demonstrated the anatomical configurations that determine access pain or neural irritation in patients with LDH [23,24]. Other researchers recommend using surgical tips for reducing pain during the procedure [20,25]. Regarding common intracanal (subarticular or central) LDH, the transforaminal approach can be conducted through Kambin’s triangle or a designated safe working zone [1,26]. However, the endoscopic approach for foraminal or extraforaminal LDH may be more challenging [27]. The rate of significant access pain during TELD is reportedly higher in patients with foraminal LDH than that in individuals with paramedian LDH [27]. Mechanically, herniated disc fragments typically block or deviate Kambin’s triangle. Chemically, the landing point is inflamed and sensitized. Therefore, the ENR is vulnerable to instrumental manipulation, which may cause significant access pain. Severe access pain may delay surgical approach and lead to failure. Understanding the factors that contribute to pain during TELD can help surgeons prevent or manage pain before and during the procedure, potentially improving patient outcomes. To our knowledge, no reliable predictive rules for access pain have been established for foraminal or extraforaminal LDH.

This study aimed to predict access pain during TELD for foraminal or extraforaminal LDH and discuss pain prevention and management before and during the procedure. We hypothesized that certain anatomical configurations and patient factors could predict access pain during TELD for foraminal or extraforaminal LDH.

## 2. Materials and Methods

### 2.1. Study Design and Patients

**Figure 1:**
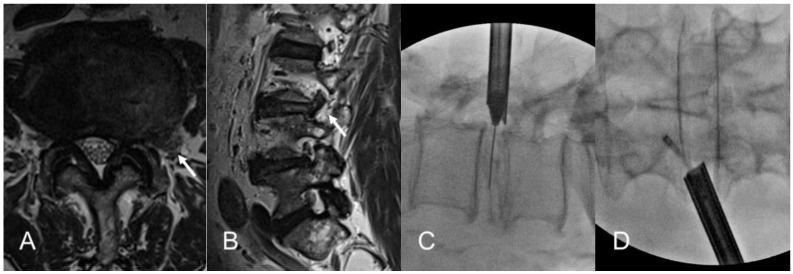
Illustrates the case of an 82-year-old male patient with intractable left leg radicular pain. Preoperative magnetic resonance imaging reveals a left extraforaminal LDH (arrows) at the L3–4 level (**A**,**B**). The patient underwent TELD under local anesthesia. The fluoroscopic image shows docking of the working sheath and exploration of the foraminal and extraforaminal zones (**C**,**D**). Despite severe access pain, the radicular pain subsided; the global clinical outcome is rated as excellent. LDH, lumbar disc herniation; TELD, transforaminal endoscopic lumbar discectomy.

This observational study included 73 patients who underwent TELD for foraminal or extraforaminal LDH between January 2017 and December 2022. Patient data were prospectively registered; their records were retrospectively evaluated. Our Institutional Review Board approved the study (GDIRB2023-210, 24 June 2023); written informed consent was obtained from the patients. The inclusion criteria were patients with single-level LDH despite >6 weeks of nonoperative treatment or those experiencing unbearable pain and progressive motor deficits. Enrollment of patients with foraminal or extraforaminal LDH was determined based on the LDH zone [28]. Patients with subarticular or central LDH were excluded from the study. Other exclusion criteria comprised severe central stenosis, segmental instability (including spondylolisthesis), massive cauda equina syndrome, inflammatory disease, infectious disease, and spinal neoplasm. The radiculopathy symptoms were compatible with both the magnetic resonance imaging (MRI) and computed tomography (CT) scan findings (Figure 1A,B).

### 2.2. Surgical Procedure

#### 2.2.1. Patient Preparation

The endoscopic procedure was performed under local anesthesia with conscious sedation according to the standard TELD technique [3,20,27,29]. Premedication included intramuscular administration of midazolam (0.05 mg/kg) and intravenous administration of fentanyl (0.8 μg/kg). Additional fentanyl doses were administered as necessary, depending on the patient’s vital signs and sedation level. The patients were then positioned prone on a radiolucent spine table.

#### 2.2.2. Transforaminal Approach Under Fluoroscopic Guidance

An 18 G needle was percutaneously inserted posterolaterally with approximately a 45º angle under fluoroscopic guidance. The approach angle and insertion point were determined based on the body size and zone of the LDH. The primary goal of this posterolateral approach was safe landing close to the herniated disc fragment with minimal access pain. The needle was inserted into the disc through the foraminal window to prevent ENR irritation after preemptive epidural block. Intraoperative discography was performed using contrast medium and indigo carmine to stain the nucleus and herniated fragments. Subsequently, a sequential dilation technique was used until the working sheath was docked at the foraminal zone, outside or inside the disc surface (Figure 1C,D).

#### 2.2.3. Selective Discectomy Under Endoscopic Visualization

**Figure 2:**
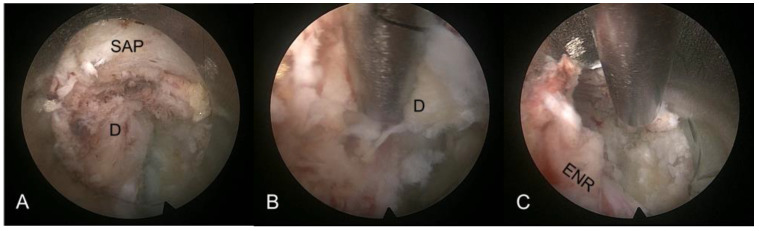
Intraoperative endoscopic images of TELD for extraforaminal LDH (L3–4, left). (**A**) The hidden exiting nerve root (ENR) is compressed and adhered to the herniated disc fragments (D). (**B**) Selective discectomy can be performed with dissection and release of tissue adhesions. (**C**) Final view showing the released ENR. Free mobilization of the ENR can determine the endpoint. LDH, lumbar disc herniation; TELD, transforaminal endoscopic lumbar discectomy; SAP, superior articular process.

An oval-shaped working channel endoscope was introduced through the working sheath, initiating selective discectomy under endoscopic visualization. Decompression was performed by visualizing anatomical structures from the posterolateral aspect. The initial view included the foraminal disc surface, perineural fat, and ENR course. The herniated disc fragment and neural tissues could be visualized and distinguished using instrumental dissection with a probe, forceps, and a radiofrequency tip. The herniated fragment compressing the nerve root usually adhered to the tenacious fibrotic annular anchorage. After delicately releasing the annular anchorage, the herniated disc fragment was freed and removed using grasping forceps and radiofrequency. During selective discectomy, epidural bleeding or inflamed tissues were controlled using radiofrequency coagulation and hemostatic agents. As the dissection and removal maneuvers proceeded, the nerve root and dural sac were visualized and released. The entire fragment, including the hidden intradiscal portion, was removed. Any remaining fragment of the “iceberg” might cause incomplete decompression or postoperative recurrence. Sufficient annular release and removal of the entire herniated disc were primary keys to success (Figure 2).

#### 2.2.4. Postoperative Management

The endpoint of the procedure was determined by free mobilization and pulsation of the nerve root and dural sac after sufficient discectomy. After the procedure, the patients were checked for adverse events before discharge. Postoperative MRI or CT scans could be considered as required.

### 2.3. Evaluation

Patient demographic data, including age, sex, body mass index (BMI), and symptom duration, were documented. Preoperative radiographic information included the level, zone (foraminal or extraforaminal), degree of disc herniation (bulging or diffuse herniation, protrusion, extrusion, and sequestration), and presence of neural anomalies (conjoined nerve root or low-lying nerve root). The foraminal nerve root impingement grade was measured using the Lee classification system [30,31].

The primary outcome was access pain, which was prospectively evaluated in all patients. Access pain was defined as mechanical or neural pain experienced during the transforaminal approach under local anesthesia. Such pain may have been caused by irritation of the ENR by the approaching needle, dilators, or working sheath when they touched or passed through a foraminal window. The intensity of the access pain during TELD was classified into a four-point scale according to a published article [27]: (1) minimal (no or negligible irritation response), (2) mild (mild but tolerable, visual analog scale (VAS) 1–3), (3) moderate (definitive complaint of pain, VAS 4–6), and (4) severe (screaming and twisting in pain, VAS > 6). Moderate or severe pain was considered “significant”.

Operative data, including operative time, length of hospital stay, and adverse events, were documented as secondary outcomes. The clinical outcomes were assessed using patient-based outcome questionnaires. These questionnaires were administered during outpatient office visits and telephone interviews. Global clinical outcomes were evaluated using the modified MacNab criteria [3,32].

### 2.4. Statistical Analysis

Analyzing the association between access pain and other variables, the independent *t*-test was performed for continuous variables; the chi-square test or Fisher’s exact test was performed for categorical variables.

Simple multiple logistic regression analysis was conducted to analyze the effects of the significant variables on access pain and the impact of access pain on clinical outcomes, including the modified MacNab criteria and complications. Simple multiple linear regression analysis was performed to analyze the effect of access pain on the operative time and hospital stay. Statistical analysis was conducted using SPSS (version 22.0; IBM Corp., Armonk, NY, USA) and R 4.3.1 (R Foundation for Statistical Computing, Vienna, Austria), with two-sided tests performed at a significance level of 5%.

## 3. Results

### 3.1. Demographics

The study comprised 73 patients with a mean follow-up period of 31.6 months (range: 12–64 months). Among them, 23 (31.51%) were male and 50 (68.49%) were female patients, with a mean age of 61.05 years (range: 21–83 years). Evaluation based on the four-point classification of transforaminal access pain revealed no or minimal pain in forty-six (63.01%) patients, mild pain in seventeen (23.29%), moderate pain in six (8.22%), and severe pain in four (5.48%). Therefore, the rate of significant (moderate to severe) access pain was 13.70% (10 of 73 patients). The zones of disc herniation were foraminal in 53 patients (72.60%) and extraforaminal in 20 (27.40%). The operative level was L2–3 in three (4.11%), L3–4 in twelve (16.44%), L4–5 in twenty-nine (39.73%), and L5–S1 in twenty-nine (39.73). The mean operative time was 62.71 min (range, 30–120 min). The mean length of hospital stay was 2.29 days (range, 1–9 days). Evaluation based on the modified MacNab criteria indicated excellent outcomes in twelve patients (16.44%), good outcomes in forty-seven (64.38%), fair outcomes in eleven (15.07%), and poor outcomes in three (4.11%). The symptomatic improvement and success (good or excellent) rates were 95.89% and 80.82%, respectively. Postoperative dysesthesia was observed in six patients (8.22%), managed through medication alone or in combination with nerve root block. No instances of postoperative infection or hematoma were reported. Three patients with poor outcomes underwent revision surgery (decompression with fusion) during follow-up. The demographic characteristics are summarized in Table 1.

### 3.2. Predictive Factors for Access Pain During TELD

Extraforaminal LDH tended to cause more severe pain than did foraminal LDH during the transforaminal approach under local anesthesia (*p* < 0.001). Univariate analysis revealed a significant association between the herniation zone and access pain. Significant access pain occurred in 5.66% of foraminal LDH cases and 35% of extraforaminal LDH cases (*p* = 0.0032; Table 1). Other factors including age, sex, BMI, symptom duration, level, side, herniation type, and foraminal stenosis grade were not associated with access pain (Table 1). According to the multiple logistic regression analysis, the herniation zone was also strongly associated with significant access pain (odds ratio = 6.264; 95% confidence interval = 1.056–37.164; *p* = 0.043; Table 2).

### 3.3. Access Pain and Clinical Outcomes

Analysis of the impact of access pain on operative results showed that access pain affected operative time and hospital stay. Significant access pain tends to prolong operative time and length of hospital stay. The mean operative time was 59.57 ± 16.21 min in the minimal pain group and 82.50 ± 26.06 min in the significant pain group (*p* < 0.001; Table 3). The mean length of hospital stay was 2.10 ± 1.42 days in the minimal pain group and 3.50 ± 2.51 days in the significant group (*p* = 0.012; Table 3). Access pain did not affect other outcome parameters, including complications, postoperative dysesthesia, and the modified MacNab criteria (Table 3). Excellent or good outcomes were observed in 82.54% of the minimal pain group and 70% of the significant pain group, showing no statistical difference.

Multiple linear regression analysis showed that significant access pain was strongly associated with a longer operative time (B = 21.839; standard error = 6.707; *p* = 0.002; Table 4). Significant access pain was also associated with a longer hospital stay (B = 1.106; standard error = 0.518; *p* = 0.036; Table 5).

## 4. Discussion

### 4.1. Importance of Access Pain During TELD Under Local Anesthesia

TELD has emerged as an efficient surgical alternative to treating LDH by using a percutaneous transforaminal endoscopic approach. Its effectiveness has been validated in randomized trials and meta-analyses [7,8,9,10,11,12,13,14,15,16,17,18,19]. Endoscopic spine surgeons can perform this technique with the typical benefits of minimally invasive procedures, including muscle preservation and the avoidance of unnecessary laminofacetectomy under local anesthesia. A direct posterolateral approach to the spinal canal through the foraminal safety zone and selective discectomy are feasible for typical intracanal (central or subarticular) LDH. However, the transforaminal approach may encounter considerable access pain in cases of foraminal or extraforaminal LDH, stemming from nerve root irritation, potentially jeopardizing the procedure’s success [27]. Therefore, prediction and prevention strategies for access pain are essential for aspiring endoscopic spinal surgeons.

### 4.2. Higher Risk of Access Pain for Extraforaminal LDH

In our study, the rate of significant access pain was 13.70% (10 of 73 patients). It was relatively lower than the rate of 24% (6 of 25 patients) found in previously published data [27].

**Figure 3:**
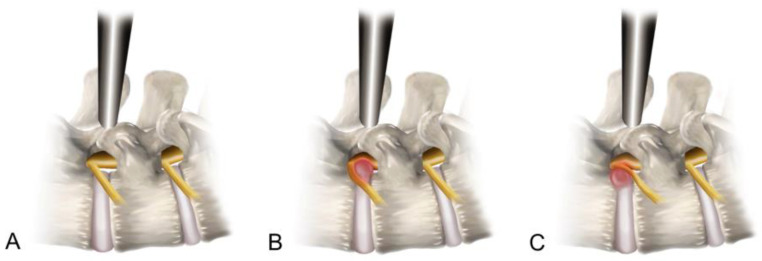
Schematic comparison of the transforaminal approach for foraminal and extraforaminal LDH. In the normal lumbar segment (**A**), the safety working zone is indicated. Regarding foraminal LDH, the safety working zone may be inflamed but preserved for the transforaminal approach (**B**). By contrast, the safety working zone may be narrowed in extraforaminal LDH and consequently cause considerable ENR irritation and access pain during the transforaminal approach (**C**). ENR, exiting nerve root; LDH, lumbar disc herniation.

Our data revealed a significant association between the herniation zone and access pain experienced during the transforaminal approach. Extraforaminal LDH, whether with or without sequestered fragments, tended to cause significant access pain (*p* < 0.01). We postulated that this correlation was closely related to the safety zone of the transforaminal approach (Figure 3). In cases of foraminal LDH, as the disc material extrudes, the ENR usually deviates anteriorly, allowing for the maintenance or widening of the Kambin’s triangle space utilized in the transforaminal approach. However, in instances of extraforaminal LDH, the extruded disc material can directly exert lateral pressure on the ENR. Therefore, the ENR can be located in the middle of the approach trajectory, leading to a subsequent narrowing of the safety zone. Under such circumstances, the risk of experiencing significant nerve root irritation pain may increase, even with a careful outside-in approach.

### 4.3. Access Pain May Prolong Operative Time and Hospital Stay

In our study, access pain during TELD tended to lead to longer operative time (*p* < 0.01) and hospital stay (*p* < 0.05). However, other clinical outcomes, including the modified MacNab criteria, postoperative dysesthesia, and other complications, were not significantly associated with access pain during TELD. However, caution should be exercised when interpreting these findings. First, the skill and experience of the operating surgeon can influence the outcomes of endoscopic spinal procedures. The senior surgeon involved in this study possessed extensive expertise in transforaminal endoscopic spine surgery, potentially mitigating surgical failures or complications. The observed outcomes may not be generalizable to cases handled by less-experienced practitioners. Second, patients may retain painful memories, impacting their long-term satisfaction, regardless of pain scores or functional improvements. Therefore, access pain may affect both global clinical outcomes and recovery time. Proactive measures to predict and minimize access pain during TELD under local anesthesia are essential for achieving clinical success.

### 4.4. Technical Keys to Avoid Access Pain

The primary goal of the transforaminal endoscopic approach is to dock the obturator and working sheath in the foraminal zone while avoiding ENR irritation. Accomplishing this objective may involve employing specific technical insights and strategies.

First, the outside-in approach is superior to the inside-out technique. The herniated disc fragment tightly compresses the nerve root. Therefore, passing through the disc may increase the probability of nerve root irritation. In contrast, adopting an outside-in approach can minimize neural irritation or damage during the approach process.

Second, implanting a preemptive epidural block in the foraminal zone can reduce pain caused by neural irritation. Several authors have reported the efficacy of preemptive blocks during the transforaminal endoscopic approach [22]. The administration of block medications may widen the working space.

Third, the landing point should be as far away from the ENR as possible. The target is recommended to be located at the caudal part of the disc to avoid ENR. Regarding Kambin’s triangle, the safe working space at the caudal level of the disc was larger than that at the cranial level.

Fourth, a serial dilation technique, starting from thin to larger dilators, may also reduce mechanical pain and create a smooth route in the back muscles. In contrast, blunt pressure caused by a large-headed dilator may cause severe pain on the inflamed disc surface.

Fifth, using a bevel-ended working sheath is more valuable than using a non-bevel-ended working sheath. The sloping edge enables precise foraminal landing while protecting the ENR. To avoid ENR irritation, the sharp edge of the working sheath is directed towards the caudal part of the foramen. During this step, the obturator and working sheath should not be inserted into the disc space because blunt insertion of the devices can cause severe neural damage or irritation.

Finally, the working sheath can be engaged in the bony foramen with delicate mallet tapping. This allows the working sheath to be placed firmly without a handgrip; the percutaneous transforaminal approach can be completed. A working channel endoscope can then be introduced through the working sheath for adequate decompression.

### 4.5. Limitations of the Study

This study has some inherent limitations. First, despite the inclusion of consecutive cases, its retrospective nature may have introduced considerable bias in assessing the effect of access pain on long-term clinical outcomes and patient satisfaction. Second, in addition to ENR irritation, the severity of access pain might be influenced by other factors, such as personal disposition, depth of sedation, premedication, and the surgeon’s skill. Our measurement was confined to the global pain response of the patients, preventing the evaluation of potential additional factors. Third, the grade of access pain was determined arbitrarily using a four-point grading system. An agreement study on the degree of access pain was not conducted. Therefore, our subsequent study will focus on developing a more objective and reliable grading system for access pain or nerve irritation during the percutaneous transforaminal approach under local anesthesia through a prospective study. Furthermore, future studies should evaluate the relationship between the radiographic dimensions of Kambin’s safety zone and access pain or neural irritation.

## 5. Conclusions

TELD can serve as an effective surgical option for treating foraminal/extraforaminal LDH using the percutaneous transforaminal approach under local anesthesia. This local procedure may be beneficial, particularly in medically compromised or older patients. However, access pain during the transforaminal approach can result in surgical failure and other adverse events. In our study, significant access pain developed during TELD for extraforaminal LDH compared with foraminal LDH, leading to prolonged operative time and length of hospital stay. The extraforaminal component of LDH can narrow the safety working zone for the transforaminal approach. Thus, addressing specialized technical considerations is imperative to ensure the clinical success of TELD.

## Figures and Tables

**Table 1:** Relationship between preoperative factors and access pain during TELD.

Items		Total (*N* = 73)		Access Pain				*p*-Value
				Minimal (*n* = 63)		Significant (*n* = 10)		
Age (years), mean, SD		61.05	10.58	61.49	8.96	58.30	18.23	0.599
Sex	Male	23	31.51%	20	31.75%	3	30.00%	1.000
	Female	50	68.49%	43	68.25%	7	70.00%	
BMI (kg/m^2^), mean, SD		23.64	3.55	23.56	3.61	24.20	3.26	0.598
Symptom Duration	Acute	8	10.96%	5	7.94%	3	30.00%	0.072
	Subacute	10	13.70%	10	15.87%	0	0	
	Chronic	55	75.34%	48	76.19%	7	70.00%	
Level	L2–3	3	4.11%	3	4.76%	0	0	1.000
	L3–4	12	16.44%	10	15.87%	2	20.00%	
	L4–5	29	39.73%	25	39.68%	4	40.00%	
	L5–S1	29	39.73%	25	39.68%	4	40.00%	
Side	Lt	38	52.05%	33	52.38%	5	50.00%	1.000
	Rt	35	47.95%	30	47.62%	5	50.00%	
Herniation Type	Extruded	63	86.30%	53	84.13%	10	100%	0.433
	Protruded	9	12.33%	9	14.29%	0	0	
	Bulging, diffuse	1	1.37%	1	1.59%	0	0	
Herniation Zone	Foraminal	53	72.60%	50	79.37%	3	30.00%	0.003 **
	Extraforaminal	20	27.40%	13	20.63%	7	70.00%	
Grade (foraminal stenosis)	1	8	10.96%	8	12.70%	0	0	0.700
	2	24	32.88%	20	31.75%	4	40.00%	
	3	41	56.16%	35	55.56%	6	60.00%	

TELD, transforaminal endoscopic lumbar discectomy; BMI, body mass index; SD, standard deviation; * *p* < 0.05, ** *p* < 0.01.

**Table 2:** Impact of herniation zone on access pain. Results of logistic regression analysis (simple/multiple regression analysis).

Items		Unadjusted Model (Simple Regression)			Adjusted ^‡^ Model (Multiple Regression)		
		OR ^¶^	95% CI ^ψ^	*p*-Value	OR ^¶^	95% CI ^ψ^	*p*-Value
Herniation zone	Foraminal	Ref	-	-	-	-	-
	Extraforaminal	8.974	2.035–39.573	0.004 **	6.264	1.056–37.164	0.043 *

OR ^¶^, odds ratio; CI ^ψ^, confidence interval; ^‡^ regression models adjusted for age, sex, body mass index, symptom duration, level, side, herniation type, and grade of foraminal stenosis; * *p* < 0.05, ** *p* < 0.01.

**Table 3:** Relationship between access pain during TELD and outcome data.

Items		Total (*N* = 73)		Access Pain				*p*-Value
				Minimal (*n* = 63)		Significant (*n* = 10)		
Operative Time (min), mean, SD		62.71	19.34	59.57	16.21	82.50	26.06	<0.001 **
Hospital Stay (days), mean, SD		2.29	1.66	2.10	1.42	3.50	2.51	0.012 *
Postop Dysesthesia	No	67	91.78%	58	92.06%	9	90.00%	1.000
	Yes	6	8.22%	5	7.94%	1	10.00%	
Modified MacNab	Excellent	12	16.44%	11	17.46%	1	10.00%	0.611
	Good	47	64.38%	41	65.08%	6	60.00%	
	Fair	11	15.07%	9	14.29%	2	20.00%	
	Poor	3	4.11%	2	3.17%	1	10.00%	

TELD, transforaminal endoscopic lumbar discectomy; SD, standard deviation; * *p* < 0.05, ** *p* < 0.01.

**Table 4:** Impact of access pain on prolongation of the operative time. Results of linear regression analysis (simple/multiple regression analysis).

Items		Unadjusted Model (Simple Regression)				Adjusted ^‡^ Model (Multiple Regression)			
		B ^¶^	SE ^ψ^	t	*p*-Value	B ^¶^	SE ^ψ^	t	*p*-Value
Access pain	Minimal	Ref	-		-	-	-		-
	Significant	22.929	6.046	3.792	<0.001 **	21.839	6.707	3.256	0.002 **

B ^¶^, non-standardized coefficient; SE ^ψ^, standard error; ^‡^ regression models adjusted for age, sex, body mass index, symptom duration, level, side, herniation type, herniation zone, and grade of foraminal stenosis; * *p* < 0.05, ** *p* < 0.01.

**Table 5:** Impact of access pain on prolongation of the length of hospital stay. Results of linear regression analysis (simple/multiple regression analysis).

Items		Unadjusted Model (Simple Regression)				Adjusted ^‡^ Model (Multiple Regression)			
		B ^¶^	SE ^ψ^	t	*p*-Value	B ^¶^	SE ^ψ^	t	*p*-Value
Access pain	Minimal	Ref	-		-	-	-		-
	Significant	1.405	0.545	2.578	0.012 *	1.106	0.518	2.136	0.036 *

B ^¶^, non-standardized coefficient; SE ^ψ^, standard error; ^‡^ regression models adjusted for age, sex, body mass index, symptom duration, level, side, herniation type, herniation zone, and grade of foraminal stenosis; * *p* < 0.05, ** *p* < 0.01.

## Data Availability

The data presented in this study are available on request from the corresponding author.
